# Editorial: The evolution and sustainability of societal systems

**DOI:** 10.3389/fsoc.2025.1636379

**Published:** 2025-06-27

**Authors:** Juan R. Coca, Susana Gómez-Redondo, Juan A. Roche Cárcel

**Affiliations:** ^1^University of Valladolid, Soria, Spain; ^2^University of Alicante, Sant Vicent del Raspeig, Spain

**Keywords:** social evolution, social change, comprehension, semiotic, hermeneutic

The concept of social evolution is fundamental to understanding how social changes, based on the above elements just described, have shaped the current structures of our societies. The idea of social evanescence (Marx, M. Berman), which refers to the ephemeral and fragile nature of current social structures, presents a significant challenge to the concept of social evolution. In a global world marked by crises the question arises as to whether we can create mechanisms or strategies that promote social sustainability. Let's see how the articles in this monographic issue of Frontiers have done so, which we have structured into the following thematic blocks.

## Sociological aspects

Beriain et al. takes a sociological look at the concept of evolution in social analysis, understood in a non-teleological way, and challenges the traditional ideas of thinkers such as Comte, Spencer and Parsons, who defended a linear and progressive view of social development. The authors stress that contemporary perspectives find in the processes of cultural transmission and in the tensions between different forms of social change a more faithful understanding of social phenomena. They examine how Weber introduces a conception of modernity characterized by the tension between disenchantment and re-enchantment of the world, which breaks with the notion of a unidirectional advance toward science and progress. The article suggests that we should understand social evolution as a multiple, plural and constantly tense process.

Labora González and Fernandez-Vilas delves into how the COVID-19 pandemic has radically changed our social, cultural, economic and political life around the world. In this regard, the authors argue that we should view the pandemic as a “total social fact,” a term introduced by Marcel Mauss and continued by Durkheim, meaning that complex social phenomena impact all aspects of people's lives in a given society. Indeed, COVID-19 has left traces in health, economics, politics, culture, social interrelationships and social cohesion, suggesting that society, as a whole, could be affected in the future by significant transformation. COVID-19 is not only a biological challenge, as it has also highlighted and exacerbated existing social inequalities and disproportionately affected the most vulnerable communities and individuals.

Aguiluz-Ibargüen's text explores the semantic origin of the transformations that the relationship between Nature and Culture has undergone. In some contexts, this relationship is presented as a continuum, while in others it is shown as a well-defined duality between the two spheres. The condition that makes this argument possible is analyzed on the basis of a narrative that begins to objectify “nature” within the new modern cosmology that emerged in the seventeenth century, influenced by the contributions of Galileo and Descartes. Sociological evidence is presented that challenges the validity of this separation between Nature and Culture. The resurgence of the nature-culture continuum is examined in the context of a new late modern cosmology.

Fan's article delves into the theoretical approaches of multiculturalism, social capital and sustainability by exploring social sustainability in contexts where cultural diversity is key. To do so, he used a two-stage methodological design: in the first, a systematic review of 210 academic publications was conducted, from which conceptual elements were extracted to help define new constructs at the micro level: individual social inclusion, individual social capital and individual social sustainability. In the second phase, he conducted a textual analysis of 130 short interviews (with a total of 173 participants). This study also provides an innovative perspective by highlighting the role of micro social inclusion and individual social capital in everyday life as essential drivers of social sustainability.

Buribayev et al. explores how values have changed in Kazakh society, focusing on youth and their connection to past generations. An interesting duality is highlighted: young people still value family and community, but, at the same time, they are increasingly inclined toward individualism, self-expression, and personal success. Indeed, the findings demonstrate that while the majority of young people consider family to be their most important value, there is a notable decline in the relevance they ascribe to other traditional values, such as work commitment and religion. The study reveals that Kazakh youth are experiencing an increase in personal wellbeing and satisfaction with life, even though the importance of friendships and religion has declined.

## Educational aspects

Nasri and Souid explores how Physical Education teachers play a crucial role in the socialization of students in schools and how this discipline not only focuses on the development of physical skills, but also promotes the acquisition of verbal, relational and emotional competencies, which are key to social integration. It also mentions that socialization through physical education can positively influence how students view themselves and others, helping them face challenges such as respect for diversity and inclusion. The article highlights that Physical Education teachers, being role models, have a great responsibility in transmitting values and encouraging positive behaviors, which contributes to social cohesion and the comprehensive development of adolescents.

Francisco Carrera's article delves into the fascinating connection between Transhumanism, Education and Hermeneutics. He proposes an approach that seeks to balance technological advancement with the preservation of essential human values. Carrera argues that today's education needs to adapt to the speed of knowledge in this digital age, without forgetting the achievements of the past. The author suggests that education should be an interpretive process in which educators act as guides who help students navigate between traditional knowledge and the new realities that are emerging.

Gómez-Redondo and Obregón focuses on the need for a more flexible and adapted pedagogy that fosters social and educational sustainability in different contexts. Based on the notion of authors such as Van Mannen (or, further back in time, Herbart), they propose “pedagogical tact” (the ability of educators to adjust to changing classroom situations) and “edusemiotics” (the study of signs and meanings in education) as fundamental tools for promoting sustainable education. This perspective requires teacher training that values the interpretation and adaptation of educational cues, as well as a deep understanding of socio-cultural contexts.

## Final coda

This monographic issue has emphasized the social and educational changes experienced by contemporary societies in specific countries of the world, although its approach can be assumed to be general and universal. By focusing on structural variations, it has made it possible to confirm not only the validity and importance of the social evolutionary approach, but also its multidimensional character.

